# Validation of Quantitative Structure-Activity Relationship (QSAR) Model for Photosensitizer Activity Prediction

**DOI:** 10.3390/ijms12128626

**Published:** 2011-11-29

**Authors:** Neni Frimayanti, Mun Li Yam, Hong Boon Lee, Rozana Othman, Sharifuddin M. Zain, Noorsaadah Abd. Rahman

**Affiliations:** 1 Department of Chemistry, Faculty of Science, University of Malaya, Lembah Pantai 50603, Kuala Lumpur, Malaysia; E-Mails: nenifrimayanti@yahoo.com (N.F.); smzain@um.edu.my (S.M.Z.); 2 Drug Design and Development Research Group, University of Malaya, Lembah Pantai 50603, Kuala Lumpur, Malaysia; 3 Drug Discovery Group, Cancer Research Initiatives Foundation, Sime Darby Medical Centre, Subang Jaya, Selangor Darul Ehsan 47500, Malaysia; E-Mails: munli.yam@carif.com.my (M.L.Y.); hongboon.lee@carif.com.my (H.B.L.); 4 Department of Pharmacy, Faculty of Medicine, University of Malaya, Lembah Pantai 50603, Kuala Lumpur, Malaysia; E-Mail: rozanaothman@um.edu.my

**Keywords:** QSAR, photodynamic therapy, photosensitizer, porphyrin, IC_50_ half maximal inhibitory concentration

## Abstract

Photodynamic therapy is a relatively new treatment method for cancer which utilizes a combination of oxygen, a photosensitizer and light to generate reactive singlet oxygen that eradicates tumors via direct cell-killing, vasculature damage and engagement of the immune system. Most of photosensitizers that are in clinical and pre-clinical assessments, or those that are already approved for clinical use, are mainly based on cyclic tetrapyrroles. In an attempt to discover new effective photosensitizers, we report the use of the quantitative structure-activity relationship (QSAR) method to develop a model that could correlate the structural features of cyclic tetrapyrrole-based compounds with their photodynamic therapy (PDT) activity. In this study, a set of 36 porphyrin derivatives was used in the model development where 24 of these compounds were in the training set and the remaining 12 compounds were in the test set. The development of the QSAR model involved the use of the multiple linear regression analysis (MLRA) method. Based on the method, *r*^2^ value, *r*^2^ (*CV*) value and *r*^2^ prediction value of 0.87, 0.71 and 0.70 were obtained. The QSAR model was also employed to predict the experimental compounds in an external test set. This external test set comprises 20 porphyrin-based compounds with experimental IC_50_ values ranging from 0.39 μM to 7.04 μM. Thus the model showed good correlative and predictive ability, with a predictive correlation coefficient (*r*^2^ prediction for external test set) of 0.52. The developed QSAR model was used to discover some compounds as new lead photosensitizers from this external test set.

## 1. Introduction

Cancer is a dangerous disease in which cells grow and divide beyond their normal limits. Currently, the major treatments for cancer include surgery, chemotherapy, and radiation [1]. However, high incidences of undesirable side effects have prompted researchers to search for safer and more effective treatments.

Photodynamic therapy (PDT) provides an alternative treatment for cancer with relatively low side effects [2]. This treatment uses the combined effects of light and light activated toxic drugs or photosensitizers to target tumor cells. Photosensitizers are chemical compounds that could be excited by light of a specific wavelength [3], often with visible or near infrared light. A photosensitive drug absorbs photons which alter the drugs into an excited state. These excited drugs then pass their energy to oxygen to form free radicals (singlet oxygen) which oxidize cellular structures [4–7]. Oxidative damage caused by the free radicals exceeds a threshold level causing the cells to die.

Photofrin and other early photosensitizers (often referred to as first generation sensitizers), have properties that make them less than ideal for use in clinical PDT settings. First generation photosensitizers have several serious drawbacks in that they are not specific to cancer cells, but also tend to accumulate in normal tissues [7]. This means that not only the cancer cells, but also normal cells could be damaged by the treatment. In addition, first generation photosensitizers do not discharge rapidly from the human body. Hence, patients receiving photofrin treatment must stay out of the sun for at least a month following treatment [8]. In addition, larger and deep-seated tumors cannot normally be treated with these agents.

Much work has been done to develop new photosensitizers (second generation) to improve the pharmacokinetics and physical properties of the first generation photosensitizers [9]. Important objectives for scientists remain to develop new photosensitizers of pure compounds which are activated strongly by red light above 630 nm [10].

Many QSAR approaches have been used to search for new photosensitizing agents for cancer therapy. For example, Boyle and Dolphin [11] reported the relationship between structure and properties affecting tumoricidal effects of compounds in their development of second generation photosensitizers. Henderson and co-workers [12] reported a comparative study between tumor localizing properties and hydrophilicity, as well as dimerization abilities of 28 porphyrins and pheophorbides. They observed the tumoricidal activities of the compounds to be dependent upon a delicate balance between their hydrophilic and hydrophobic characters. Another study by Potter *et al.* [13] examined the relationship between the photophysical properties and photodynamic activities of five tetrapyrroles. A good correlation between generation of singlet oxygen and PDT effect was observed. An *in vivo* structure-activity relationship of a set of silicon phthalocyanine sensitizers was reported in 1994 [14].

Henderson and co-workers [12] reported PDT activity to be a non-linear function of lipophilicity for a series of pyropheophorbide derivatives. They used a semi-empirical, non-linear activity lipophilicity relationship model, and found lipophilicity to be highly predictive for photodynamic activity. Unfortunately, accumulation of photosensitizers in the cancer tissue is not enough for good tumoricidal effects.

Another study on a QSAR model by Vanyur *et al.* [10] predicted the biological activity of a congeneric series of pyropheophorbides used as sensitizers in photodynamic therapy based on their molecular structures using multiple linear regression and artificial neural network (ANN) techniques.

In this study, QSAR models correlating the molecular characteristics of some porphyrin-based compounds with their inhibitory concentration (IC_50_) is generated. The QSAR model developed was subsequently applied to predict the PDT activity of unknown compounds, not only those in the test set (*i.e.*, data set), but also some unknown compounds used in an external test set.

## 2. Results and Discussion

### 2.1. QSAR Modeling

The best QSAR model obtained is shown below:

(1) $$\begin{array}{l} \text{Log }1/{\text{IC}}_{50}=0.96\times \text{Verloop B}2\;(\text{subst}.1)+6.43\times \text{inertia moment }3\;\text{length}-1.63\times \text{VAMP}\\ \text{octupole ZZY}+0.72 \end{array}$$

This model, developed using multiple linear regression analysis (MLRA) technique has the *r^2^* value of 0.87 and *r*^2^ (*CV*) value of 0.71. The cross-validated coefficient (*CV*) defines the goodness of prediction while the non-cross-validated conventional correlation coefficient (*r*^2^) defines the goodness of fit of the QSAR model [15]. The *F* test value is the degree of statistical confidence.

In general, a QSAR model is acceptable when it has an *r*^2^ value greater than 0.6 and *r*^2^ (*CV*) greater than 0.5 [15,16]. The *r*^2^ (*CV*) value of 0.71 exhibits a good internal predictive power of the developed model. The model also showed an *r*^2^ value of 0.87. This high value obtained added to its usefulness as a predictive tool. The statistical output of the MLRA model is presented in Table 1.

**Figure 1:**
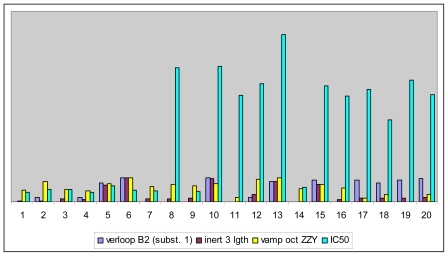
Effects of descriptors in quantitative structure-activity relationship (QSAR) model with their photodynamic therapy (PDT) activity.

Based on this QSAR model described above, it could be inferred that inhibitory activity will improve with increase of the electrostatic parameter (*i.e.*, Vamp octupole ZZY). The electrostatic parameters are properties of a molecule which are related to its electron affinity and demonstrate the susceptibility of a molecule towards attack by nucleophiles. In this study, VAMP octupole ZZY correlates well with the PDT activity. Compounds numbers 1, 2, and 5 were observed to be more active than compound numbers 11, 14, and 16 to 20. The increasing value of this descriptor (Figure 1 and Table 2) made the photosensitizers more efficiently absorb photons and produce reactive singlet oxygen (ROS). This may explain the activities observed by these photosensitizers [17,18].

Verloop parameters are sets of multi-dimensional steric descriptors. They can be used to characterize the shape and volume of the substituent, which are important in explaining the steric influence of substituents in the interactions of organic compounds with macromolecular drug receptors [19]. The verloop descriptor and PDT activity has negative correlation. Increasing value of verloop descriptor will decrease the PDT activity. Some functional groups in substituent 1 will be exerted into PDT activity, such as the presence of hydrophilic groups (*i.e.*, -COOH) and causes a decrease in the verloop values for the compounds numbers 1, 2 and 5 (0.17, 017 and 0.10, respectively); presumably resulting in the compounds being more active. Anyway, the presence of amino acid, such as in compound No. 19, increased the verloop values and decreases the PDT activity.

The QSAR model showed a negative correlation between the moment of inertia descriptor and PDT activity where molecules with smaller size and length were observed to have better PDT activities. For example, compound No. 11, which has a smaller size and length compared to compound No. 15, showed better PDT activity [20]. The statistical significance of the parameters in the QSAR model is presented in Table 3 and a brief description of these descriptors are detailed in Table 4.

**Figure 2:**
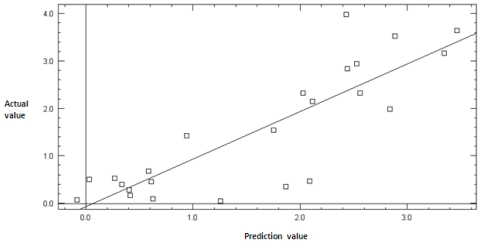
Plot of actual value *vs.* predicted value of training set.

**Figure 3:**
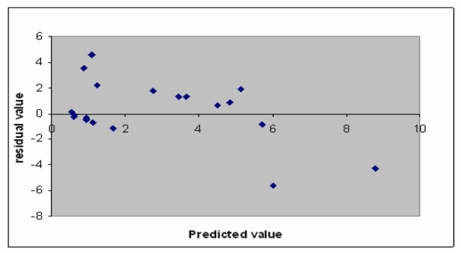
Plot of residual value *vs.* predicted value.

A plot of experimental *vs.* predicted IC_50_ is shown in Figure 2, while a plot of residual *vs.* predicted value is shown in Figure 3. These two plots are important for the predictive ability of QSAR. Residual plots (scatter) are used to detect the existence of outliers from a QSAR model [21,22]. Figure 3 shows that there are no outliers, in this study. Hence, the developed QSAR model is considered to be stable.

### 2.2. Model Validation

To determine the stability of a predictive model the most used method is by analyzing the influence of each of its elements on the final model. Any model, even with excellent goodness-of-fit and satisfactory predictions, may lack a real relationship between structural descriptors and activities. To confirm the existence of chance correlations, a reliable validation procedure must be carried out. The definitive validity of a model is examined with the external validation, to evaluate its efficacy.

The inhibition concentrations of the compounds in the test set (*i.e.*, 12 porphyrin-based compounds in the test set and 20 porphyrins-based compounds in the external test set) were predicted using the QSAR model developed in this study. The calculated IC_50_ values of the compounds in the predicted set and external test set are listed in Tables 5 and 6, respectively. The correlation coefficient (*r*^2^) between predicted and experimental value for the QSAR model was also calculated. A predictive correlation coefficient *r*^2^ value (test set) of 0.70 and external set of 0.52 were obtained for the developed QSAR model. An *r*^2^ value of more than 0.5 between the predicted and the experimental values renders the model to be good and able to predict the PDT activities of compounds not included in the model development process [21].

To further evaluate the significance of the developed model, it needs to undergo a stability test. For this, standard error of estimate and root mean squares are used. The values of standard error (*SEE*), root mean square error (*RMSE*) and root mean squares error prediction (*RMSEP*) in this model are 0.49, 3.7 and 3.6, respectively, which further adds to the statistical significance of the developed model. In addition, the low values of *SEE*, *RMSE* and *RMSEP* indicate that the developed QSAR model is stable for predicting unknown compounds in the test set.

As expected, the developed QSAR model was able to endorse the experimental IC_50_ values for the compounds in the external test set. Some of the compounds, such as **2**, **4**, **5**, **6**, and **9** are confirmed active photosensitizers; while others such as compounds **8** and **15** showed good activities in the QSAR model but did not provide good activity when tested experimentally. This difference between theoretical and experimental results may be due to the experimental conditions in which the compounds possibly did not reach the required site for action which would result in good activities. However, further experiments will have to be carried out to ascertain the reason for this inactivity.

## 3. Experimental

### 3.1. QSAR Modeling of Porphyrin

Data set of the photosensitizing agents obtained from the literature [23,24] was used to develop the QSAR models. The data set consisted of 36 chemical compounds which were divided into a training set (24 compounds) for model development and a test set (12 compounds) for model validation. In addition, 20 porphyrin-based compounds have been shown to be good photosensitizers with experimental IC_50_ values ranging from 0.39 μM to 7.04 μM. Hence, these compounds were used as external set for model validation (data shown herewith).

The training set selection was performed by first sorting through the biological activity list in increasing value. Next, the list of compounds were divided into three groups, *i.e.*, group I comprising of compounds No. 1 to 12, group II with compounds No. 13 to 24 and group III comprising of compounds No. 25 to 36. The compounds in groups I and III were assigned to the training set, and compounds in group II were assigned to the test set.

The molecular structure of each compound was sketched using ChemDraw 6.0 (Cambridge Soft) [25] and then converted to 3D using Corina in TSAR 3.3 software package (Accelrys) [26]. Cosmic in TSAR 3.3 (Accelrys) was used to optimize these molecular structures where the optimizations were terminated when the energy differences or the energy gradients become smaller than 1 × 10^−5^ or 1 × 10^−10^ kcal/mol, respectively. Molecular descriptors were also generated using TSAR 3.3 (Accelrys) [26] for each compound.

In this study, 316 descriptors were first generated for then correlation matrix was applied to select the best subset of descriptors to be included in the QSAR model development. It could be used to identify highly correlated pairs of variables, and thus identifying the redundancy in the data set. A coefficient of 1.0 indicates two variables to be perfectly correlated while a coefficient 0.0 indicates no correlation. Pair-wise correlations were performed on members of the descriptors pool, moving one of the two descriptors randomly when their correlation coefficient exceeded 0.9 [22]. The reduced descriptors pool used to develop QSAR model reported in this work contained 50 descriptors and are shown in Table 7.

The next step involved scaling the descriptors, prior to the model development stage. This was a very delicate procedure since there could be underlying relationships amongst the descriptors, and manipulations involved in this step might lead to unforeseen effects. Range scaling could assist in preventing weightings of descriptors upon the Euclidean distance calculations in multidimensional descriptors space. The scaling was calculated as follows:

(2) $$y_{i}=\frac{x_{i}-\text{min}(x)}{\text{max}(x)-\text{min}(x)}$$

where, *y_i_* is the scaled value; *x_i_* is the original value; min (*x*) is the minimum collection of *x* objects; and max (*x*) is the maximum collection of *x* objects.

### 3.2. Development of QSAR Model

The selected descriptors were then used to develop a QSAR model. In this study, the QSAR model is developed using the multiple linear regression analysis (MLRA) technique [15]. The main goal of QSAR model development is to find the best set of descriptors that will produce a stable QSAR model with the ability to predict properties of unknown compounds.

For the MLRA technique, stepwise regression was chosen in the development of the QSAR model, in which a selection algorithm was used to select a subset of the input variables, *X*. The advantage of estimating a model with stepwise MLRA is that only a few variables are needed to build the QSAR model [16]. The stepwise method combines two approaches, which are the forward and backward stepping.

In forward stepping, the partial *F* (statistical significance) values for all variables outside the model were calculated. This process is continued until no more variables qualified to enter the model. In backward stepping, the partial *F* values for all variables inside the model were calculated. The variable with the lowest partial *F* value was removed from the model. This process is continued until no more variables were qualified to be removed from the model. In general, a model can be accepted if it had fewer variables with better predictive power *r*^2^ (*CV*).

Cross validation provides a rigorous internal check on the models derived using multiple regression analysis, giving an estimate of the true predictive power of the model *i.e.*, how reliable are the predicted values for the untested compounds. The cross validation analysis in TSAR 3.3 software package (Accelrys) [26] was performed using leave-one-out method where one compound is removed from data set and its activity is predicted using the model derived from the rest of the data set [22].

### 3.3. Model Validation

The last step in QSAR model development is model validation. It is important to evaluate the robustness and the predictive capacity or validity of the model before using the model to predict and interpret biological activities of compounds in the test set. When estimating the predictive ability of QSAR models, it is necessary to distinguish two classes of predictive power, namely the internal and external predictivity. Internal predictivity measures how accurately the model can predict the bioactivities of the set of compounds (training set) used to build the statistical model. External predictivity tries to measure the predictive power for molecules to which the model has not been subjected to before. Of the two, external predictivity is observed to be more accurate [19].

In this study, external validation was performed on a test set (*i.e.*, test set and external test set). The best QSAR model developed was validated by predicting IC_50_ value of compounds in the test set, and tested for chance correlation by comparing the predicted and experimental photodynamic activities.

### 3.4. Preparation of Compounds for External Test Set

Compounds **1** and **2** were purchased from Frontier Scientific Inc., USA, and used without further purification. Experimental data for the isolation as well as the spectroscopic data for compounds **3**, **4**, **7**, **8**, and **9** have already been reported by Kamarulzaman [27], and compound **12** by Tan [28]. Compound **13** was obtained from David Appleton (Centre for Natural Product Research and Drug Discovery (CENAR), Department of Pharmacology, Faculty of Medicine, University of Malaya, Kuala Lumpur, Malaysia). The identity and purity of the compounds were confirmed using high resolution mass spectrometry (HRMS) before use. Compound **13** (aquatic samples) had been previously identified by Harris *et al.* [29].

Compounds **5** and **6** were semi-synthesized from compound **12**. Briefly, compound **12** was dissolved in *N*,*N*′-dicyclohexylcarbodiimide (DCC) and 4-dimethylaminopyridine (DMAP) in dry dichloromethane (DCM), and subsequently reacted with a mixture of H-Asp-(OtBu)(OtBu) and excess diisopropylethylamine (DIPEA) in dry dichloromethane for 3 h at room temperature. A mixture of compound **6** and unreacted compound **12** were purified using preparative thin layer chromatography (PTLC) in 7:3 hexane:acetone solvent system. The major band at R*_f_* = 0.73 was isolated, re-dissolved with cold acetonitrile, filtered and dried to yield compound **6**. The protecting group of compound **6** was removed by stirring with 1:1 ratio of DCM and trifluoroacetic acid (TFA), followed by partitioning with equal amounts of water and DCM. The organic layer was collected and dried using rotary evaporator to yield compound **5**. The identity of compound **12** as the starting material was confirmed by LCMS and UV-vis absorbance data. The structure of compound **5** was confirmed by ^1^H-NMR, HRMS and UV-vis absorbance data. The structure of compound **6** which was obtained following removal of the protecting group (OtBu) (OtBu) of compound **5** was confirmed by HRMS and UV-vis absorbance data.

Compounds **10**, **11** and **14** were isolated from a methanolic extract of the leaves of *Leonurus sibiricus*. The methanolic crude extract was purified using silica gel column chromatography, eluting with increasing amounts of acetone (0–100%) in hexane and finally with 100% methanol. Fractions 34 and 40 which were eluted at 100% acetone were combined and further purified using PTLC in 20% acetone in hexane to yield compound **11** (R*_f_* = 0.36). The band corresponding to R*_f_* = 0.55 was isolated and treated with 8:2 TFA:H_2_O to yield compound **10**. Fraction 12 which was eluted at 6:4 hexane:acetone was subjected to further purification by PTLC using 75:25 hexane:acetone to yield compound **14** (R*_f_* = 0.48). The structure of compound **10** was confirmed by HRMS and UV-vis absorbance data whereas the identity of compounds **11** and **14** was confirmed by ^1^H-NMR, HRMS and UV-vis absorbance data. The spectroscopic data of compounds **10**, **11** and **14** were in agreement with the literature data [30–32].

Compounds **15**, **16** and **17** were semi-synthesized in a similar way as compounds **5** and **6**, but with the addition of H-Lys-(OtBu)(Boc) in the reaction, instead of H-Asp-(OtBu)(OtBu). Following purification using PTLC (60:40 hexane:acetone), the major band at R*_f_* = 0.15 was isolated, re-dissolved with cold acetonitrile, filtered and dried to yield compound **15**. The protecting group of compound **15** was removed by stirring with 1:1 ratio of DCM and TFA, followed by partitioning with equal amounts of water and DCM. The organic layer was collected and dried using rotary evaporator to yield compound **16**, which was further purified using Sephadex column chromatography and 100% methanol. A second PTLC band at R*_f_* = 0.63 was isolated and treated with 1:1 DCM:TFA to yield compound **17**. The structure of compound **15** was confirmed by ^1^H-NMR, HRMS and UV-vis absorbance data. The structures of compound **16**, following the removal of the protecting group (OtBu)(Boc), and compound **17** were confirmed by HRMS and UV-vis absorbance data.

For the synthesis of compound **18**, pheophorbide-*a* was dissolved in a solution containing DCC and DMAP in dry dichloromethane. This mixture was then reacted with another mixture of H-Asp-(OtBu)(OtBu) and excess DIPEA in dichloromethane. The reaction mixture was washed, dried, and subjected to purification using silica gel column chromatography using hexane:acetone solvent system. After the major brown band was eluted from the column, the solvent was evaporated and the solid was dissolved in 100% cold acetonitrile, filtered and dried. Removal of the protecting group was performed by stirring the compound with 1:1 ratio of DCM:TFA. Following partitioning with equal amounts of water and DCM, the organic layer was collected and dried using rotary evaporator to obtain compound **18**. The structure of compound **18** was confirmed by ^1^H-NMR, HRMS and UV-vis absorbance data. Compounds **19** and **20** were semi-synthesized in a similar way as compounds **18** but with the addition of H-Lys-(OtBu)(Boc) in the reaction, instead of H-Asp-(OtBu)(OtBu). Following purification with silica gel column chromatography using hexane-acetone solvent system, the fraction corresponding to R*_f_* = 0.46 in 60:40 hexane:acetone was collected, further purified by PTLC (60:40 hexane:acetone), and subsequently treated with 1:1 DCM:TFA to yield compound **19**. Another fraction corresponding to R*_f_* = 0.42 in 60:40 hexane:acetone was collected, further purified by PTLC (60:40 hexane:acetone) and subsequently treated with 1:1 DCM:TFA to yield compound **20**. The structures of compounds **19** and **20** were confirmed by HRMS and UV-vis absorbance data.

### 3.5. Determination of Photocytotoxicity of Compounds in External Test Set by MTT (3-(4,5-dimethylthiazol-2-yl)-2,5-diphenyl-2H-tetrazolium hydrobromide) Assay

Leukemic cell line HL-60 in phenol red-free RPMI medium containing 5% fetal bovine serum were seeded in 96-well plate at the density of 15,000 cells/well. Photosensitizers, dissolved in the same medium were added at concentrations ranging from 0.01 to 10 μM. Following 2 h incubation, the cells were irradiated for 10 min with a broad spectrum light source at light dose of 5.6 J/cm^2^. The cells were further incubated for 24 h. At the end of the incubation, 20 μL of MTT solution (5 mg/mL) was added into each well and incubated for 4 h. The plate was then subjected to centrifugation at 2000 rpm for 10 min. 100 μL of medium was carefully removed and replaced with 100 μL of DMSO to dissolve the purple formazan formed. Absorbance was read at 570 nm using an OpsysMR microplate spectrometer (Thermo-Labsystems, Chantilly, VA, USA). The half maximal inhibitory concentrations (IC_50_) of the photosensitizers were then determined. Duplicate of the experiment was performed without irradiation to assess the dark toxicity of the photosensitizers. Compounds **1**–**20** showed negligible toxicity in the dark.

## 4. Conclusions

The QSAR model has been successfully developed with a good correlative and predictive ability for predicting PDT activity. This QSAR model exhibiting a high degree of accuracy was then validated by predicting the PDT activity of experimental compounds in the external test set. The PDT activity is predominantly influenced by a set of descriptors which appeared in the QSAR model such as electrostatic and steric properties. The developed QSAR model was able to discover and confirm the PDT activities of five compounds as potential active photosensitizers.

## Figures and Tables

**Table 1:** Statistical output of multiple linear regression analysis (MLRA) model.

Statistical Output	Value
Non*-*cross validated *r*^2^	0.87
Cross validation *r*^2^ (*CV*)	0.71
*F-*value	37.85
*F-*probability	1.95 × 10^−8^
Standard error of estimate *(SEE)*	0.49
Residual sum of square (*RSS*)	4.12
Predictive sum of square (*PRESS*)	9.23

**Table 2:** Descriptor values of compounds in the external test set.

No.	Verloop B2 (subst. 1)	Inert 3 Length	Vamp Octupole ZZY	Exp IC_50_ (μM)
1	0.00	0.02	0.49	0.39
2	0.17	0.03	0.85	0.52
3	0.00	0.11	0.52	0.51
4	0.17	0.11	0.44	0.39
5	0.79	0.71	0.75	0.68
6	1.00	1.00	1.00	0.50
7	0.00	0.13	0.63	0.45
8	0.00	0.13	0.73	5.63
9	0.00	0.14	0.67	0.44
10	1.00	0.98	0.77	5.69
11	0.00	0.00	0.18	4.47
12	0.17	0.29	0.93	4.96
13	0.84	0.84	0.99	7.04
14	0.00	0.00	0.56	0.62
15	0.90	0.12	0.74	4.86
16	0.00	0.09	0.58	4.45
17	0.89	0.14	0.15	4.72
18	0.80	0.14	0.29	3.43
19	0.92	0.15	0.00	5.11
20	0.97	0.17	0.30	4.49

**Table 3:** Statistical significance of parameters.

Descriptors	Regression Coefficient a	Jacknife SE b	Covariance SE c	*t*-Value d	*t*-Probability e
Verloop B2	0.96	0.41	0.44	2.16	0.05
Inertia moment 3 length	6.42	0.52	0.73	8.75	1.04 × 10^−7^
Vamp octupole ZZY	−1.63	1.06	0.80	−2.03	0.06

a The regression coefficient for each variable in the equation;

b An estimate of the standard error of each regression coefficient derived from a Jacknife procedure on the final regression model;

c Estimate of the standard error of each regression coefficient derived from the covariance matrix;

d Significance of each variable included in the final model;

e Statistical significance for t-values.

**Table 4:** Descriptors which were included in the MLRA model.

Descriptor	Symbol	Explanation
Verloop parameter	Verloop B2 (substituent 1)	The distance from the axis of the attachment bond, measured perpendicularly to the edge of the substituents.
Molecular attributes	Inertia moment 3 length	Indicates the strength and orientation behaviors of molecule in an electrostatic field.
Electrostatic parameter	Vamp octupole ZZY	Properties of molecule arising from the interaction between a charge probe, such as positive unit point reflecting a proton, and target molecule.

**Table 5:** Calculated log 1/IC_50_ for compounds in the test set.

Compounds No.	Experimental log 1/IC_50_	Predicted log 1/IC_50_
1	1.39	1.70
2	1.39	1.83
3	1.37	2.12
4	1.25	2.12
5	1.11	1.58
6	1.03	1.84
7	0.97	1.54
8	0.96	1.46
9	0.82	1.66
10	0.80	1.32
11	0.78	1.42
12	0.71	1.72

**Table 6:** Calculated IC_50_ for compounds in the external test set.

No.	Compounds	Exp. Value (μM)	Pred. Value (μM)	No.	Compounds	Exp. Value (μM)	Pred. Value (μM)
1	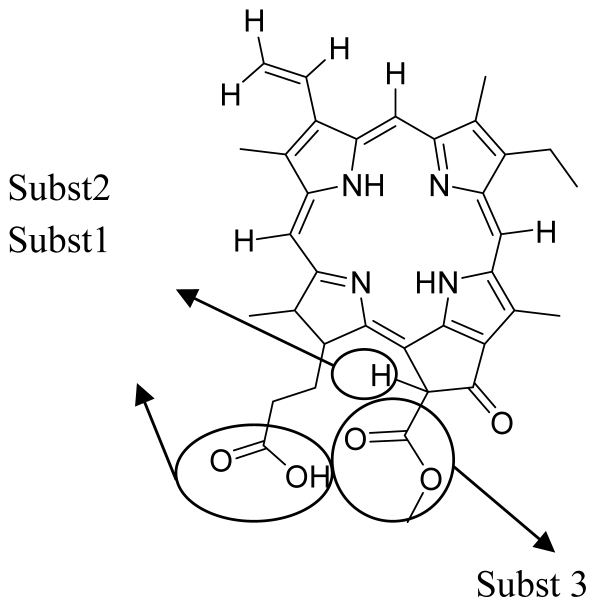 Pheophorbide A (pha)	0.39	6.02	2	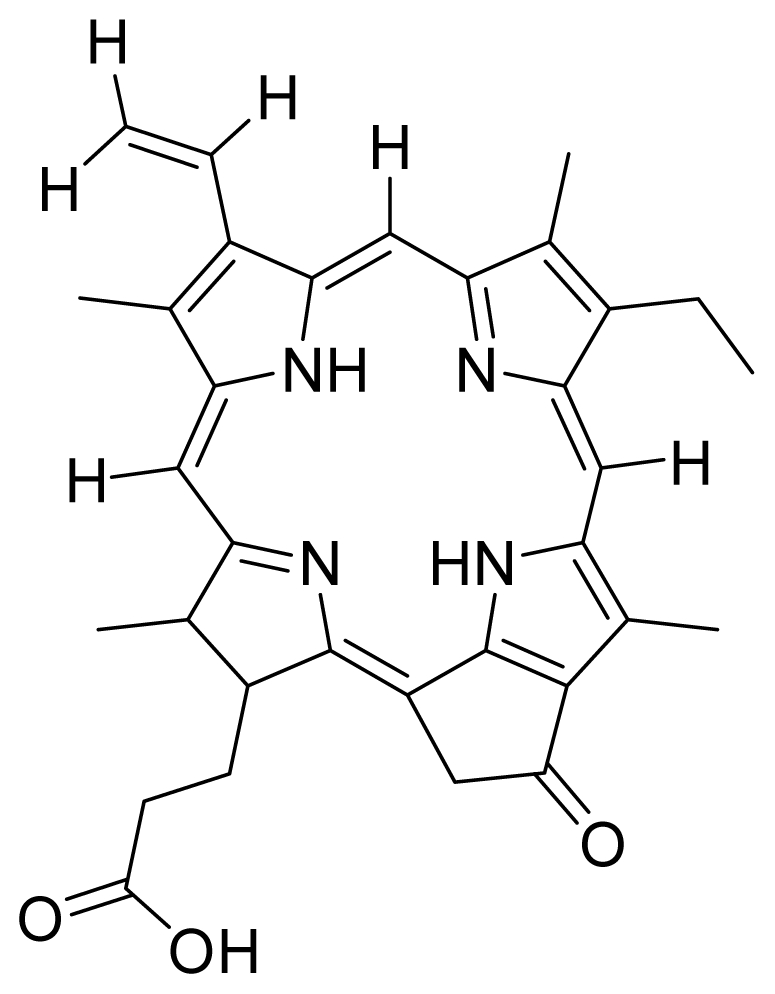 Pyropheophorbide A	0.52	0.6
3	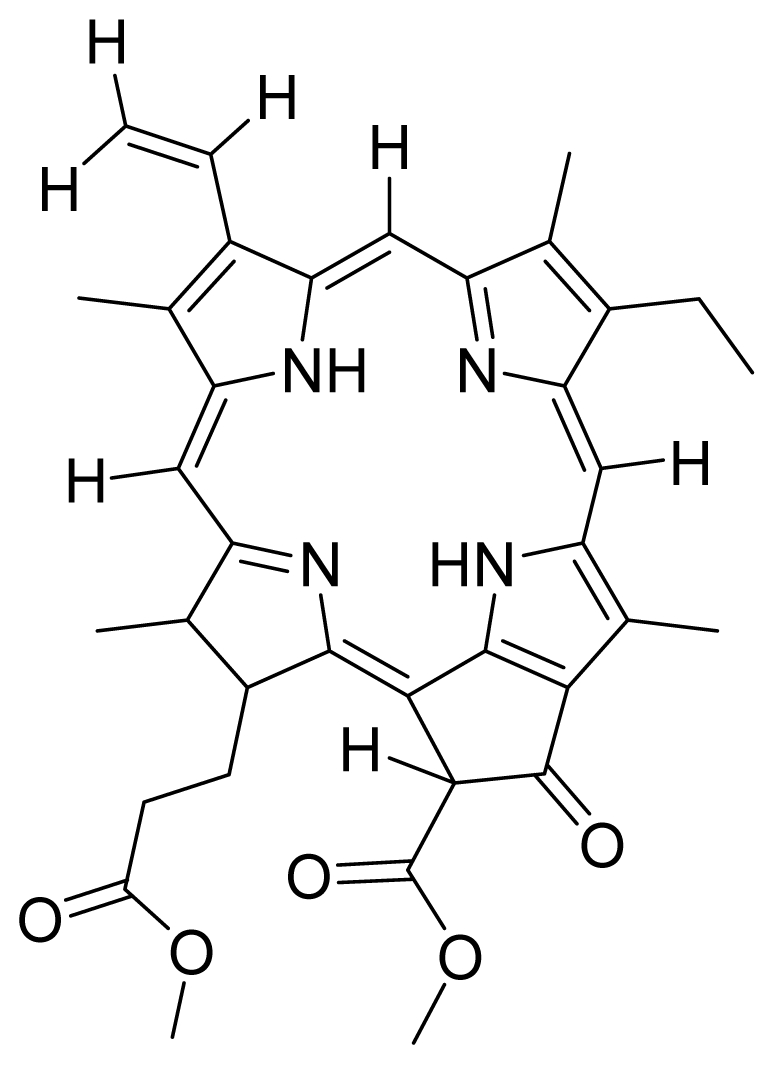 Pheophorbide A methyl ester	0.51	1.65	4	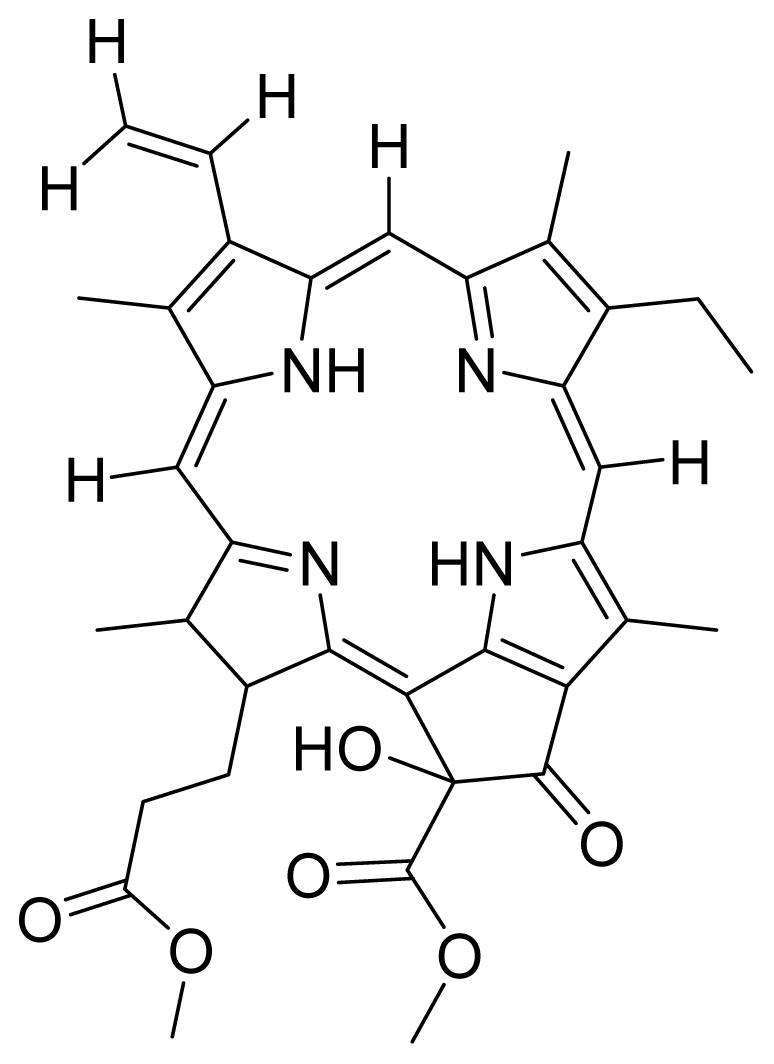 Hydroxy pheophorbide A methyl ester	0.39	0.61
5	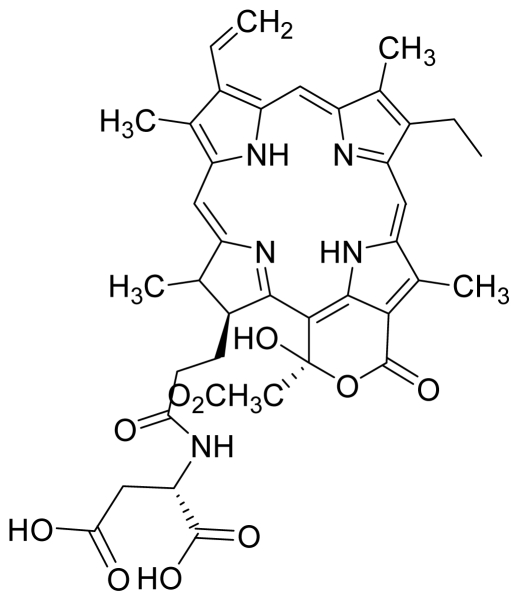 G2 aspartyl (deprotected)	0.68	0.54	6	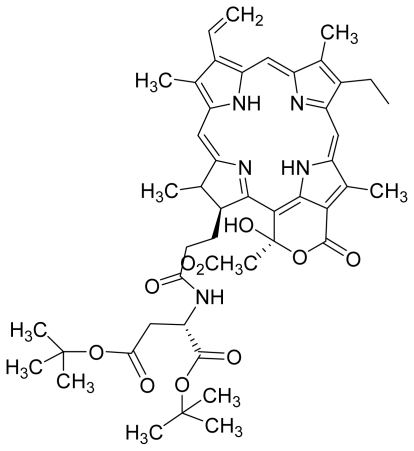 G2 aspartyl (protected)	0.50	0.56
7	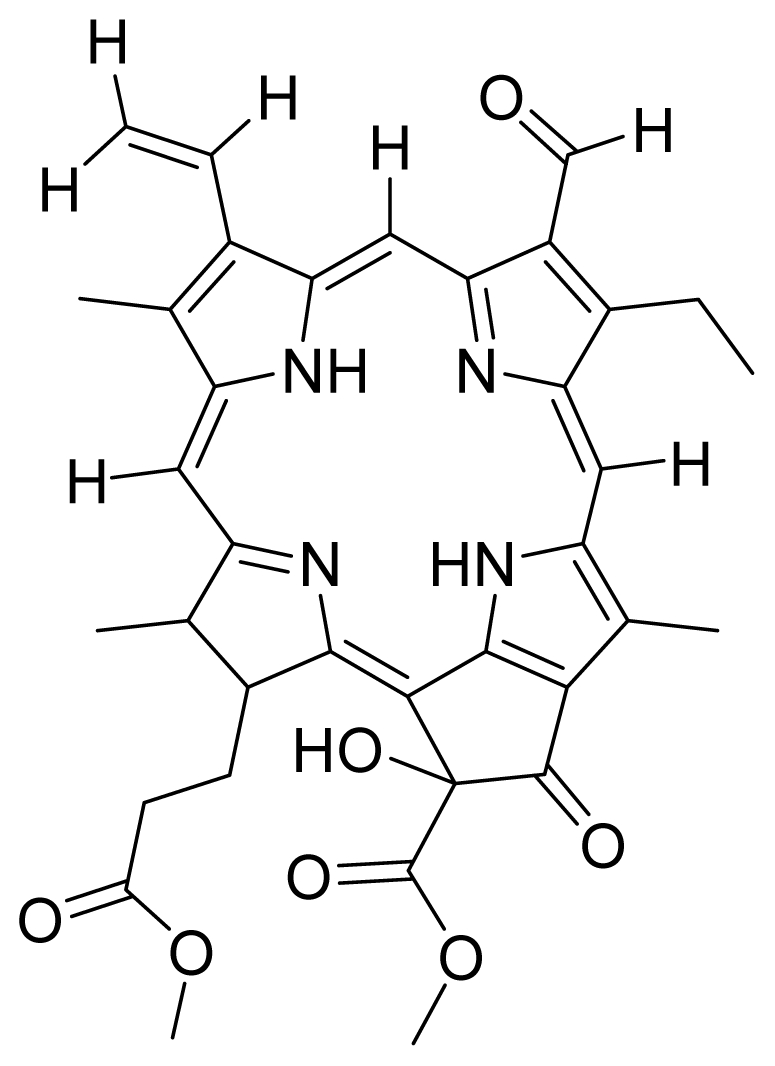 Hydroxy pheophorbide B methyl ester	0.45	1.12	8	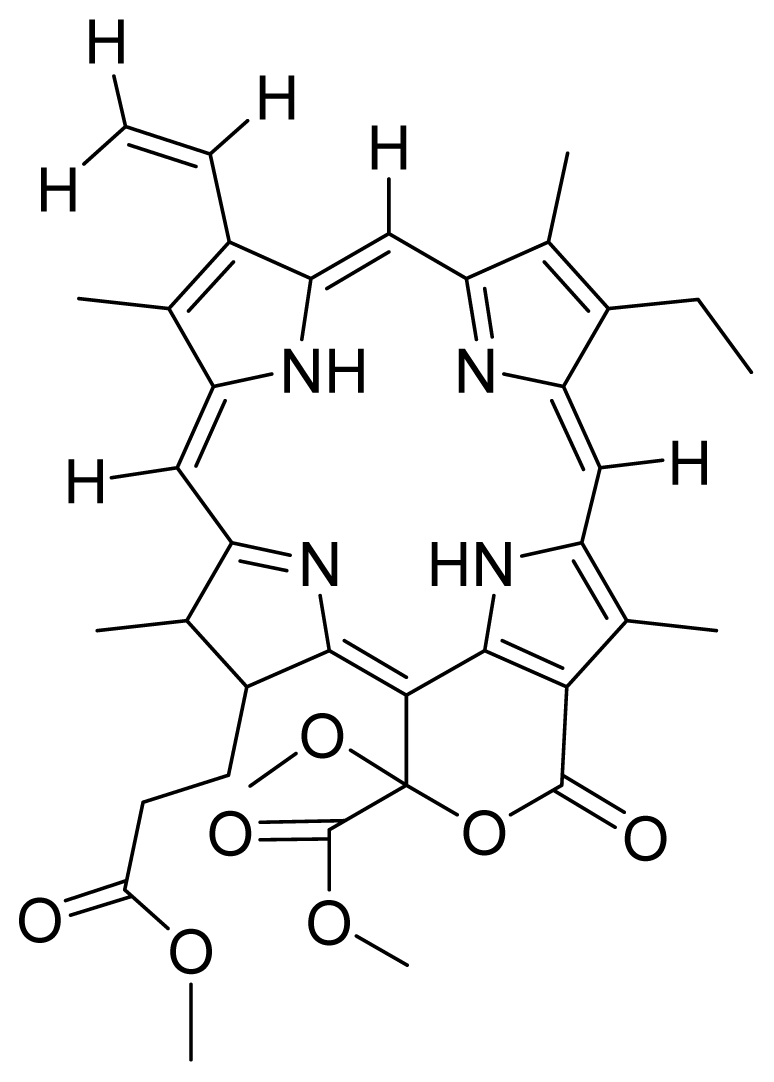 Methoxy G2 methyl ester (a type)	5.63	1.08
9	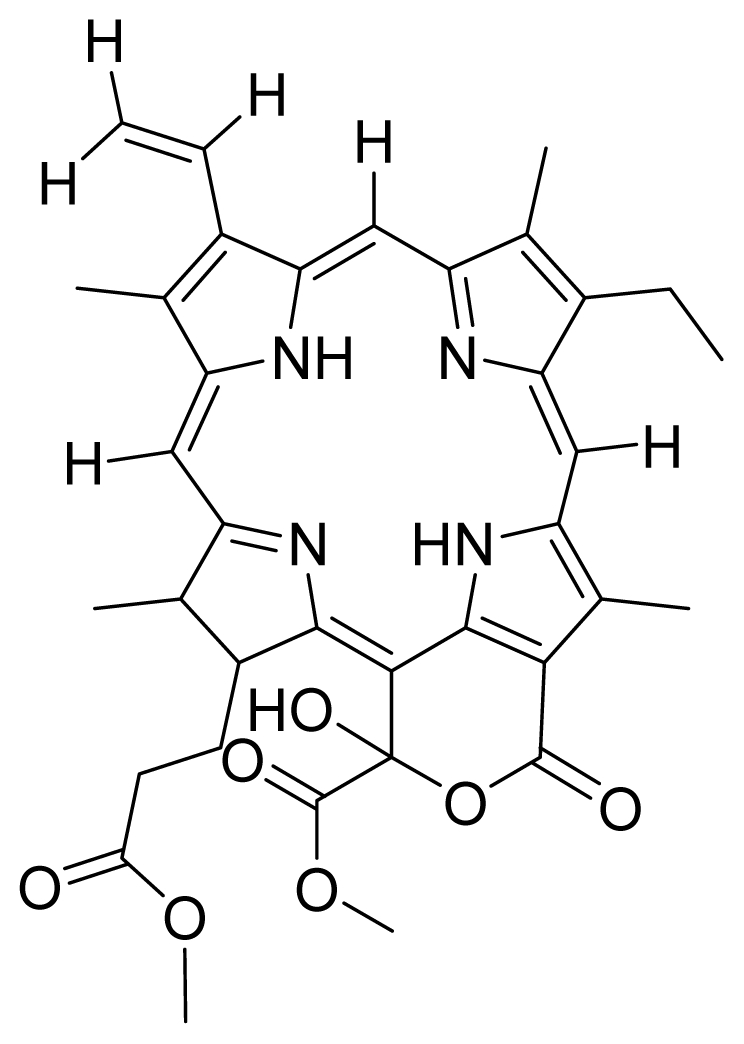 G2 dimethyl ester (15^1^- hydroxypurpurin-7-lactone methyl diester)	0.44	0.95	10	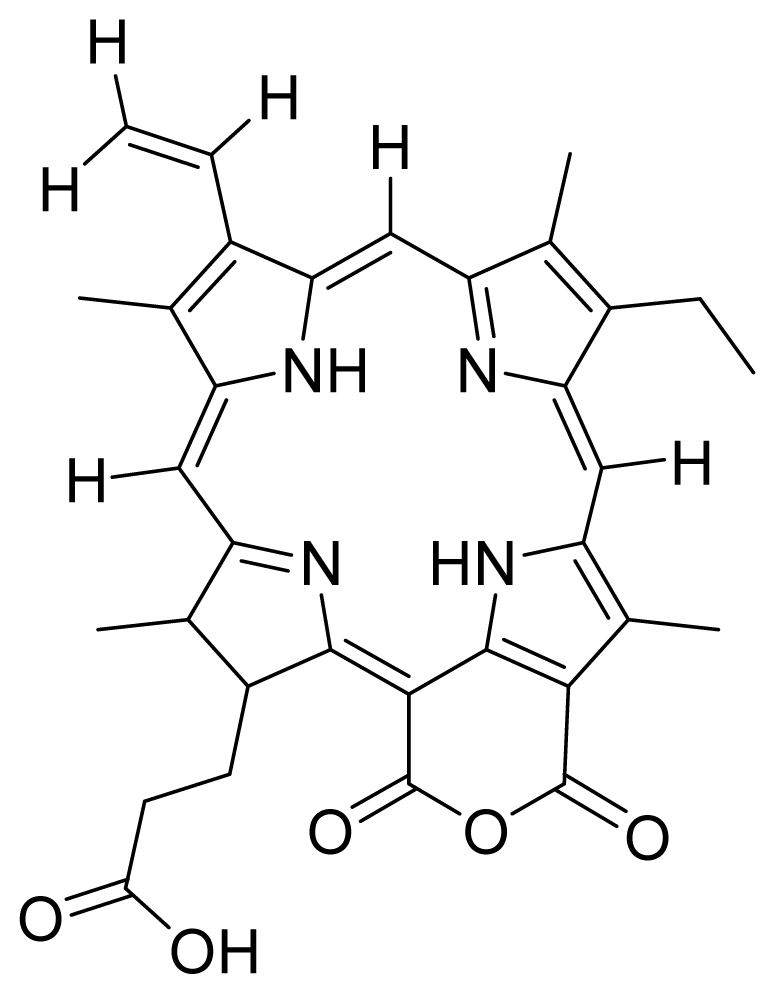 Purpurin 18 (KMP1)	5.69	4.82
11	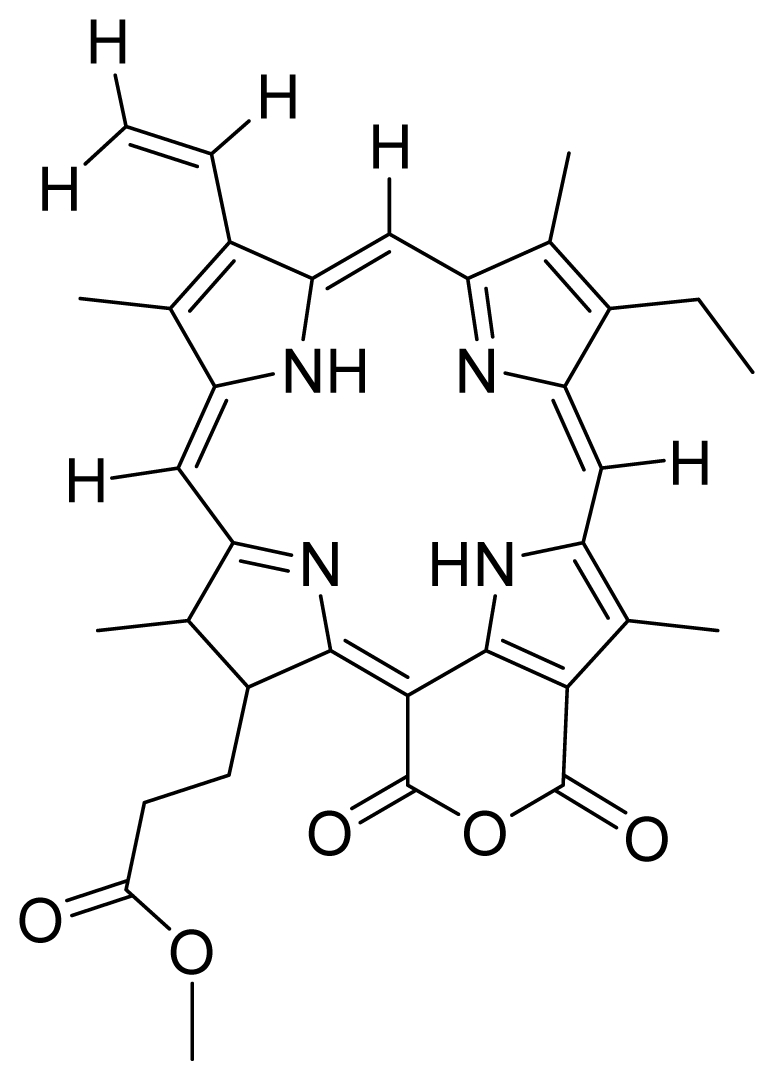 Purpurin-18 methyl ester	4.47	8.78	12	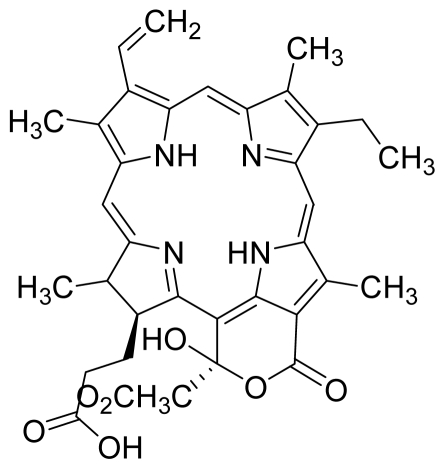 G2 acid methyl (15^1^-hydroxypurpurin-7-lactone methyl ester)	4.96	3.67
13	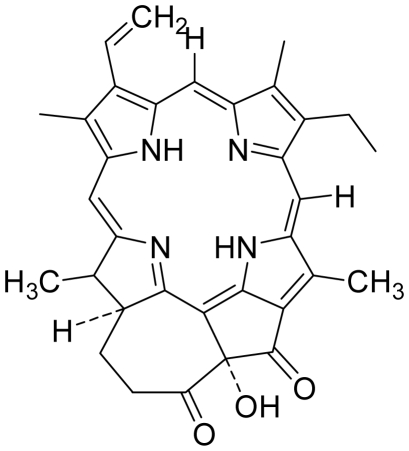 Chlorophyllone a	0.62	0.95	14	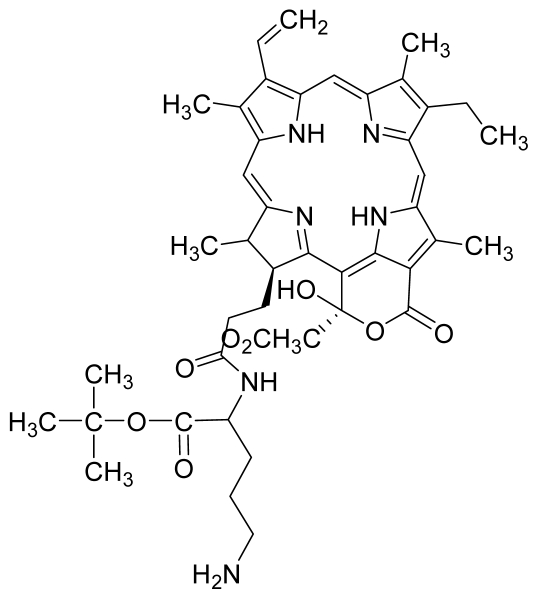 G2 lysine (protected)	7.04	5.15
15	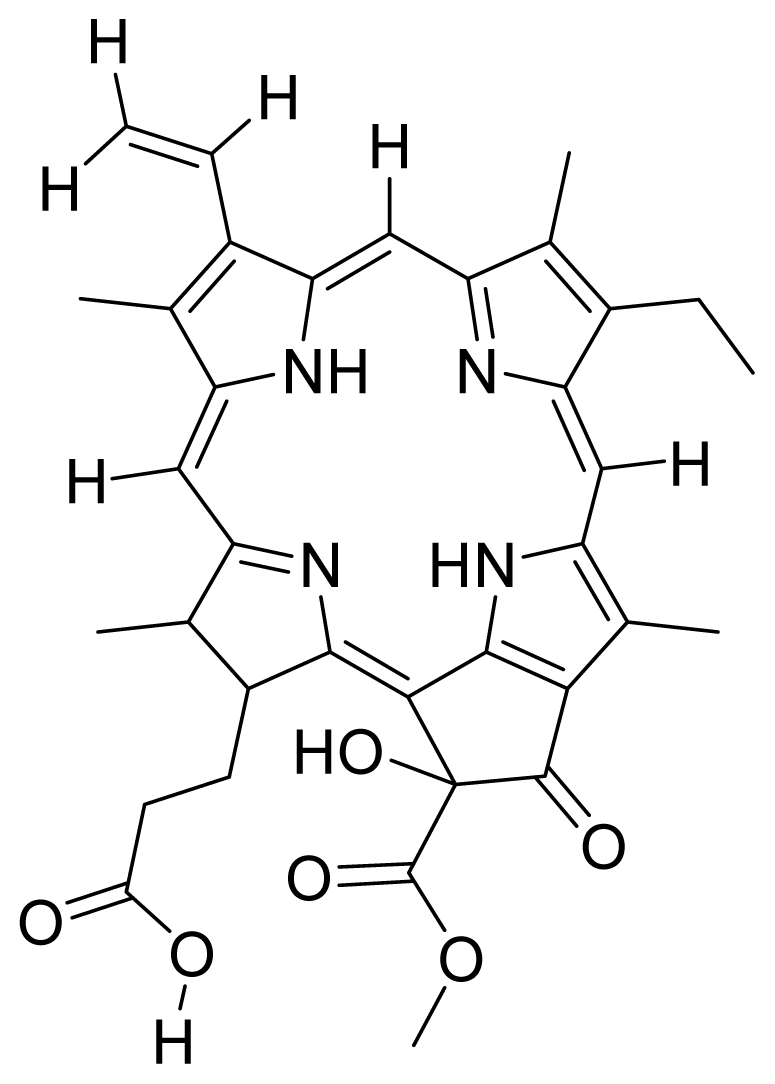 Hydroxy pheophorbide A	4.45	0.88	16	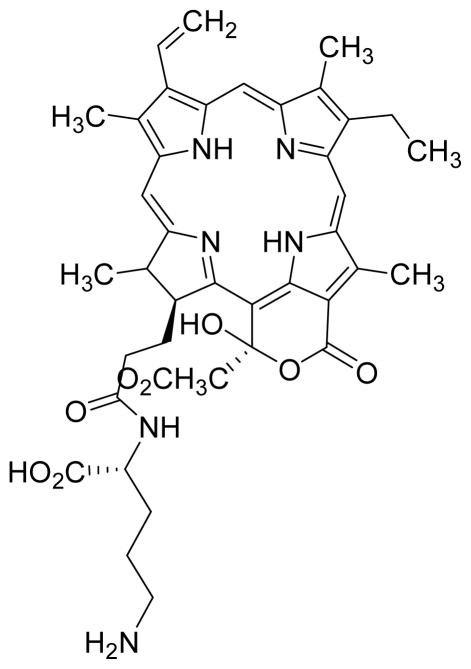 G2 lysine deprotected	4.86	5.72
17	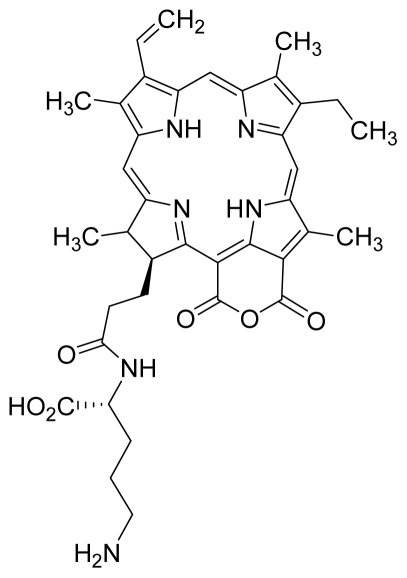 Purpurin Lys	4.72	3.43	18	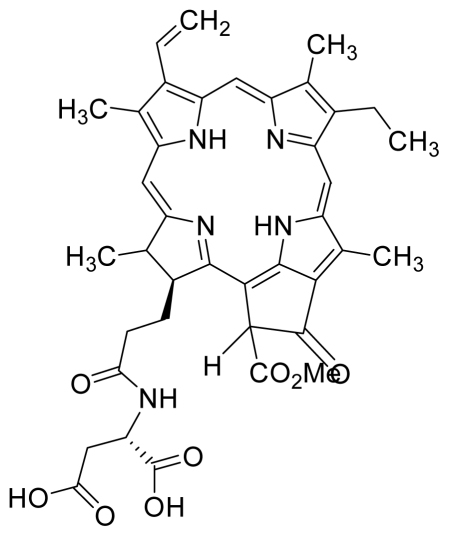 Pha Asp	3.43	1.23
19	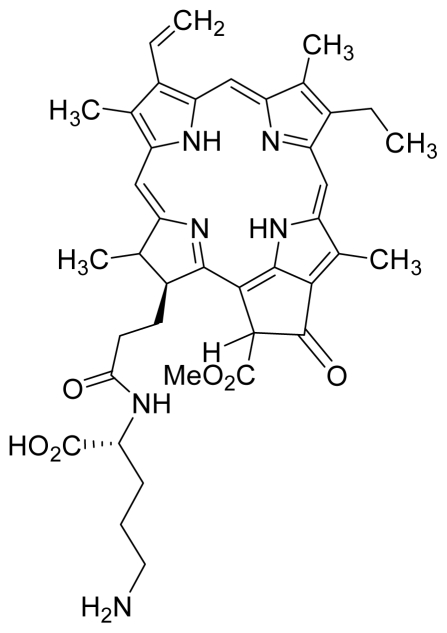 Pha Lys	5.11	4.49	20	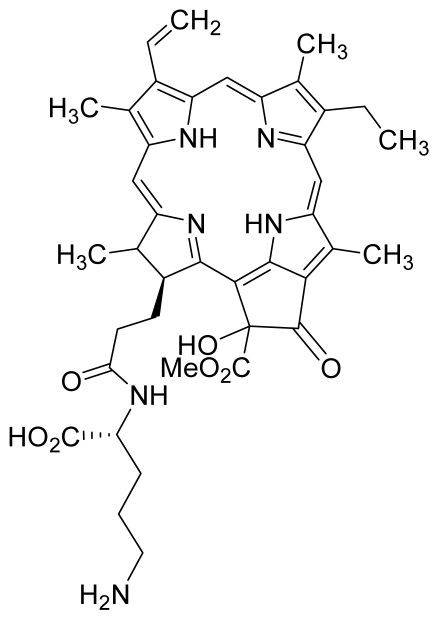 HO-Pha-Lys	4.49	2.76

**Table 7:** List of descriptors which were used to develop QSAR model.

Descriptor	Statistics		Descriptor	Statistics	
	*X̄*	SD		*X̄*	SD
Verloop L (subst. 1)	0.50	0.29	Verloop L (subst. 2)	0.23	0.26
Verloop B1 (subst. 1)	0.54	0.26	Verloop B1 (subst. 2)	0.66	0.32
Verloop B2 (subst. 1)	0.37	0.25	Verloop B2 (subst. 3)	0.05	0.11
Verloop B4 (subst. 1)	0.58	0.28	Verloop B5 (subst. 2)	0.48	0.31
Inert. Moment 2 size	0.14	0.07	Inert. Moment 1length	0.31	0.27
Inert. Moment 3 length	0.23	0.15	Ellipsoidal volume	0.15	0.07
Log P	0.60	0.26	Total lipole	0.37	0.25
Lipole X component	0.34	0.22	Lipole Z component	0.53	0.27
Kier ChiV5 (ring)	0.24	0.33	Kappa 2	0.22	0.15
Balaban topological	0.42	0.29	ADME H bond donor	0.14	0.28
ADME violation	0.27	0.27	VAMP total energy	0.78	0.15
VAMP heat of formation	0.68	0.18	VAMP HOMO	0.44	0.14
VAMP polarization XX	0.26	0.13	VAMP polarization XY	0.41	0.29
VAMP polarization XZ	0.49	0.25	VAMP polarization YY	0.33	0.16
VAMP polarization YZ	0.47	0.25	VAMP polarization ZZ	0.33	0.24
VAMP quadpole XX	0.62	0.16	VAMP quadpole XY	0.57	0.19
VAMP quadpole XZ	0.58	0.26	VAMP quadpole YY	0.55	0.18
VAMP quadpole YZ	0.34	0.21	VAMP quadpole ZZ	0.25	0.10
VAMP octupole XXX	0.14	0.09	VAMP octupole XXY	0.88	0.07
VAMP octupole XXZ	0.27	0.11	VAMP octupole YYX	0.88	0.10
VAMP octupole YYY	0.42	0.23	VAMP octupole YYZ	0.89	0.07
VAMP octupole ZZX	0.75	0.19	VAMP octupole ZZY	0.59	0.23
VAMP octupole ZZZ	0.57	0.19	VAMP octupole XYZ	0.19	0.08
Total dipole	0.26	0.16	Dipole x component	0.19	0.13
Dipole Y component	0.52	0.27	Dipole Z component	0.60	0.23

*X̄* is mean value of the descriptors; SD: standard deviation of the descriptors.
